# Cervical Ectopic Pregnancy: A Multidisciplinary Approach

**DOI:** 10.7759/cureus.19113

**Published:** 2021-10-29

**Authors:** Francisco Évora, Kristina Hundarova, Fernanda Águas, Giselda Carvalho

**Affiliations:** 1 Obstetrics and Gynecology, Coimbra Hospital and University Center, Coimbra, PRT

**Keywords:** fertility-sparing treatment, intra-amniotic injection, surgical hysteroscopy, curettage, trophoblast excision, conservative surgery, uterine artery embolization, methotrexate, uterine bleeding, cervical ectopic pregnancy

## Abstract

Cervical ectopic pregnancy is a rare but life-threatening condition in which early diagnosis and treatment are key to a successful outcome. In the past, this diagnosis led inevitably to a hysterectomy due to the risk of massive bleeding. Currently, the most effective method of treatment is yet to be found. We report a case of a 31-year-old nulliparous female with six weeks of amenorrhea and vaginal bleeding. The first approach missed the diagnosis, but an ultrasound performed by an expert revealed a gestational sac with an embryo in the cervical canal. The fertility-sparing therapeutic strategy involved performing treatment with systemic and local methotrexate, followed by embolization of the uterine artery and cervical curettage to remove the trophoblast. Our aim is to strengthen the importance of an early diagnosis and multidisciplinary perspective. Uterine artery embolization was the key to minimizing bleeding, enabling a treatment that preserved fertility.

## Introduction

Cervical ectopic pregnancy is the rarest form of ectopic pregnancy, accounting for less than 1% of these cases, with an incidence of approximately 1/8628 to 1/10000 of all pregnancies [1-3]. It is associated with high morbimortality especially if there is a delay in diagnosis or treatment, as it can complicate with profuse hemorrhage, which can lead to hysterectomy with impairment of the woman's reproductive future or even death [4]. The clinical presentation of ectopic pregnancy consists of amenorrhea and uterine bleeding with or without pelvic pain [3]. A detailed gynecological ultrasound is essential for the diagnosis of this condition. To this day, ideal management remains a matter of debate [2].

We discuss below an approach to a rare case of cervical ectopic pregnancy where medical treatment was not enough by itself to solve the situation. However, it was still possible to undertake a successful conservative surgical approach to preserve a young woman's fertility. To achieve this favorable outcome, multidisciplinary teamwork between the specialties of obstetrics, gynecology, and radiology was essential.

## Case presentation

**Figure 1:**
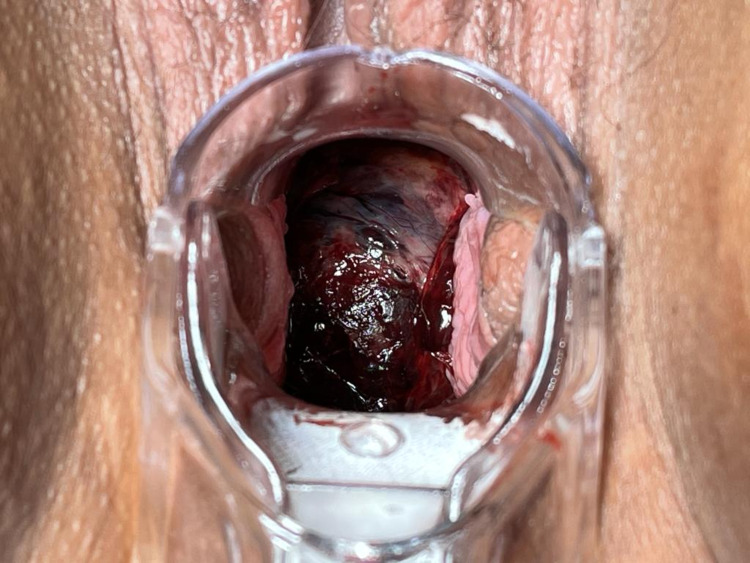
Physical examination – cervix enlarged and violet

A 31-year-old G1P0 woman with six weeks of amenorrhea and a positive urine pregnancy test presented to the obstetrics emergency department due to moderate/heavy vaginal bleeding with three days of evolution, with no pain nor other associated symptoms. She had been observed in an obstetrics consultation at the onset of symptoms and was diagnosed with an early miscarriage. It was recommended to have a follow-up ultrasound within a week. Her past medical and surgical history was irrelevant, except for laser vaporization of the cervix due to persistent low-grade squamous intraepithelial lesion. In terms of biometrics, she weighed 85 kg and was 1.72 m high (body surface of 2 m^2^). Vital signs were normal, with blood pressure of 136/81 mm Hg and pulse rate of 87 bpm. On physical examination, she presented with clots in the vagina in moderate to abundant amounts, and the cervix was enlarged and violet in color (Figure 1).

**Figure 2:**
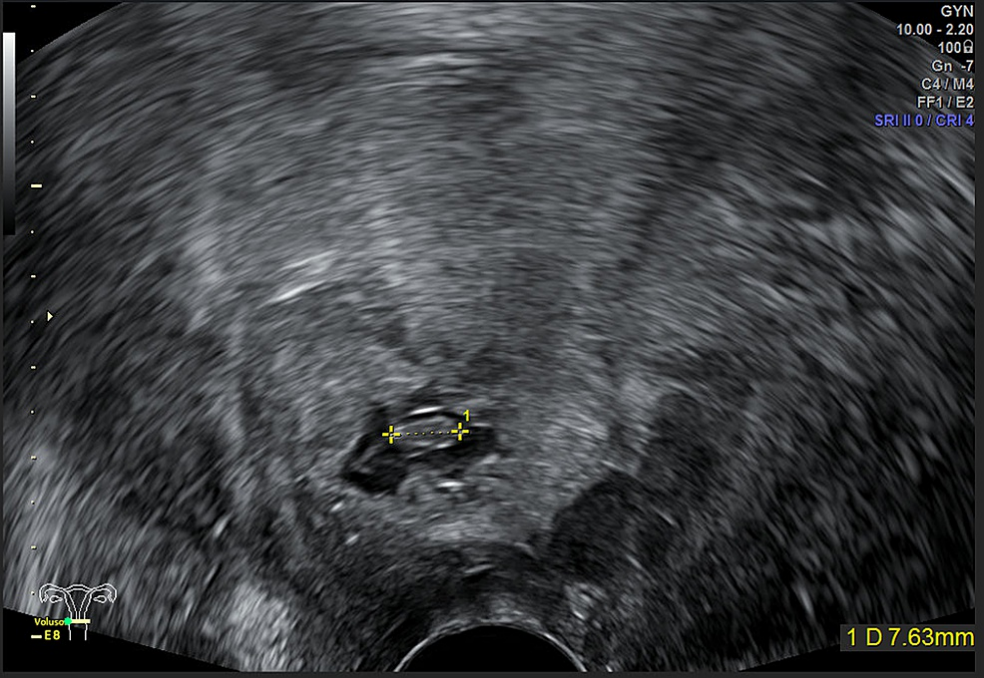
Transvaginal ultrasound (axial plane) – gestational sac in the cervical canal containing an 8 mm embryo

Transvaginal ultrasound revealed at the cervical level, in the thickness of the posterior lip, a gestational sac measuring 35 x 27 mm, containing an embryo measuring 8 mm with no cardiac activity (Figure 2).

**Figure 3:**
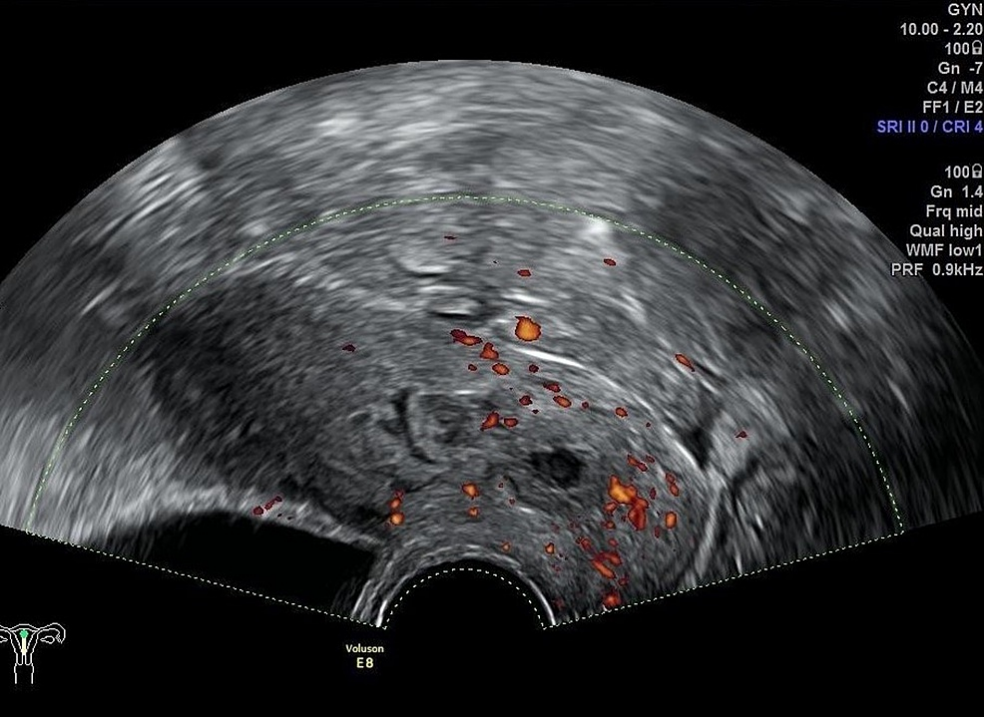
Transvaginal ultrasound (sagittal plane) – "8" or hourglass uterine shape caused by cervical enlargement

This cervical thickening caused an "8" figure of the uterus (Figure 3).

In the endometrial cavity, an anechogenic image was detected, measuring 19 mm, without trophoblastic reaction and no visible embryonic structures, suggesting intracavitary blood retention. The initial hCG value was 72.194 mIU/mL. She had mild anemia with a hemoglobin of 10.5 g/dL. Other laboratory values were within normal limits: normal platelets, coagulation tests, and liver and renal functions.

According to the diagnosis and since the patient was nulliparous and wished to preserve fertility, an inpatient conservative approach was the therapeutic option. An intramuscular dose of systemic methotrexate 100 mg (50 mg/m^2^) was initially administrated. Since the hCG values ​​remained high and the echographic dimension of the gestational sac was similar, four days after an intra-amniotic injection of methotrexate 50 mg was performed (Video 1).

**Video 1:** Intra-amniotic injection of methotrexate under transvaginal ultrasound guidance

**Figure 4:**
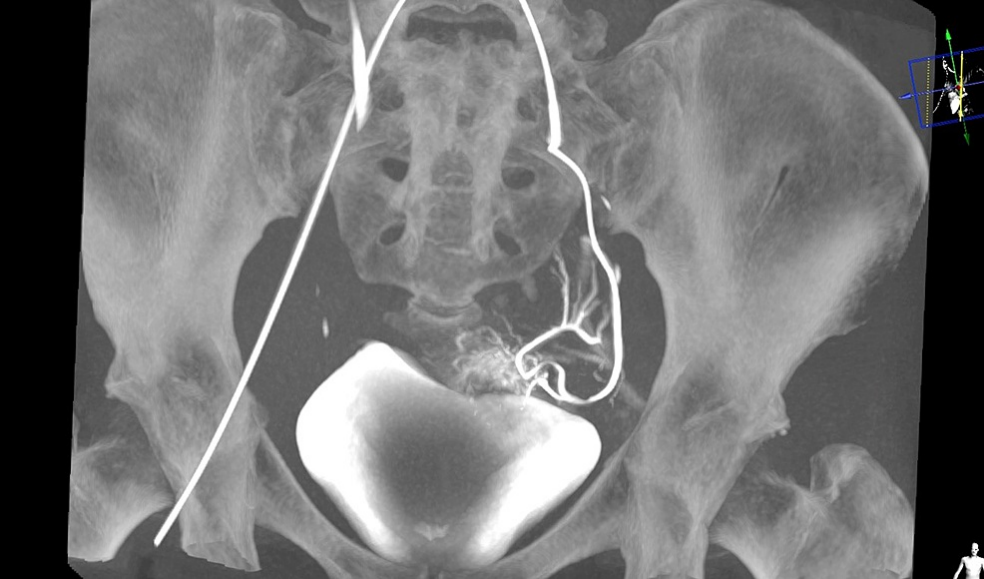
Embolization of the cervical branch of the left uterine artery

On the 12th day of hospitalization, due to worsening vaginal bleeding, selective embolization of the cervical branch of the left uterine artery was undertaken (Figure 4).

**Figure 5:**
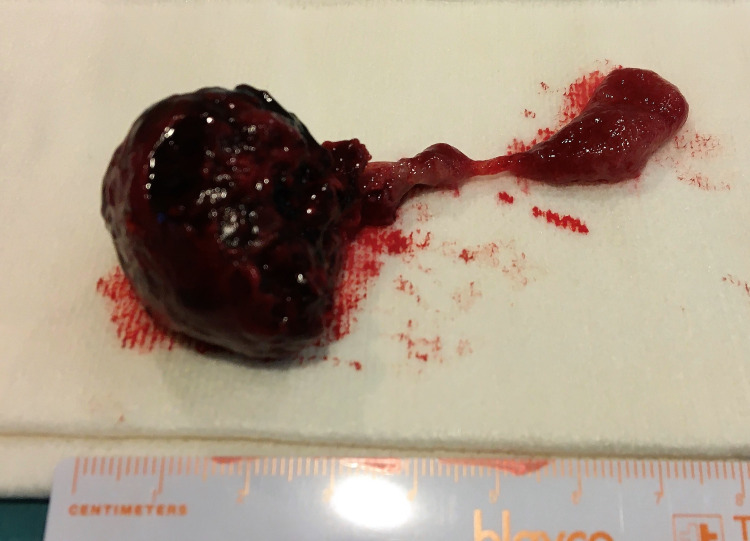
Abortion product removed by curettage

The next day, the surgical removal of trophoblastic tissue was carried out under general anesthesia. The curettage was performed under ultrasound guidance, with an easy detachment of the trophoblast and minimal blood loss. The surgical specimen was sent to the pathology laboratory (Figure 5).

**Figure 6:**
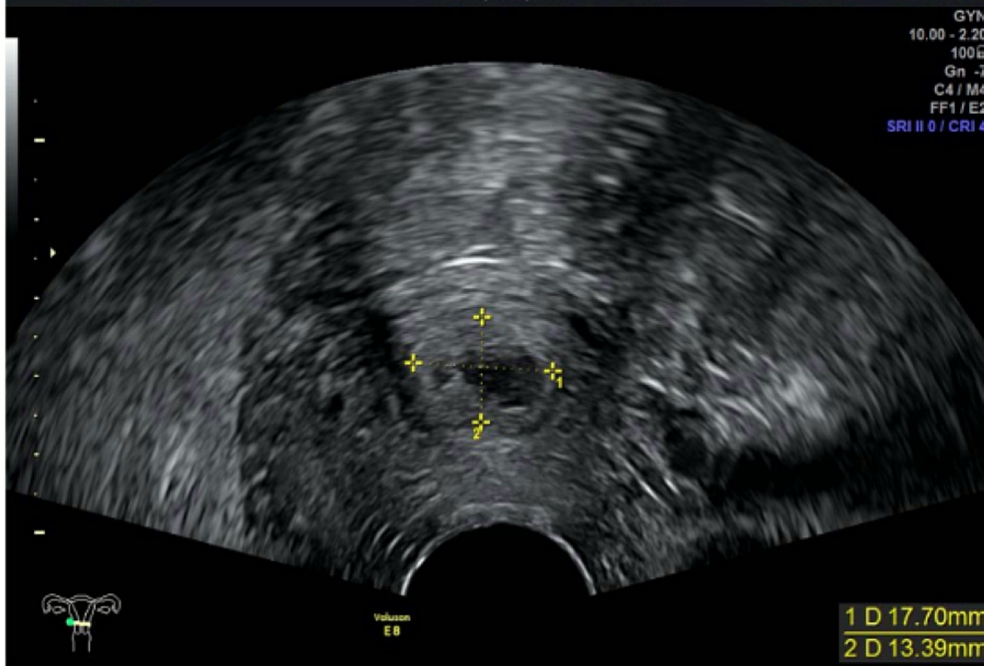
Transvaginal ultrasound (axial plane) of the residual trophoblastic tissue in resorption – nodular and cavitated image measuring 18 x 13 mm

**Figure 7:**
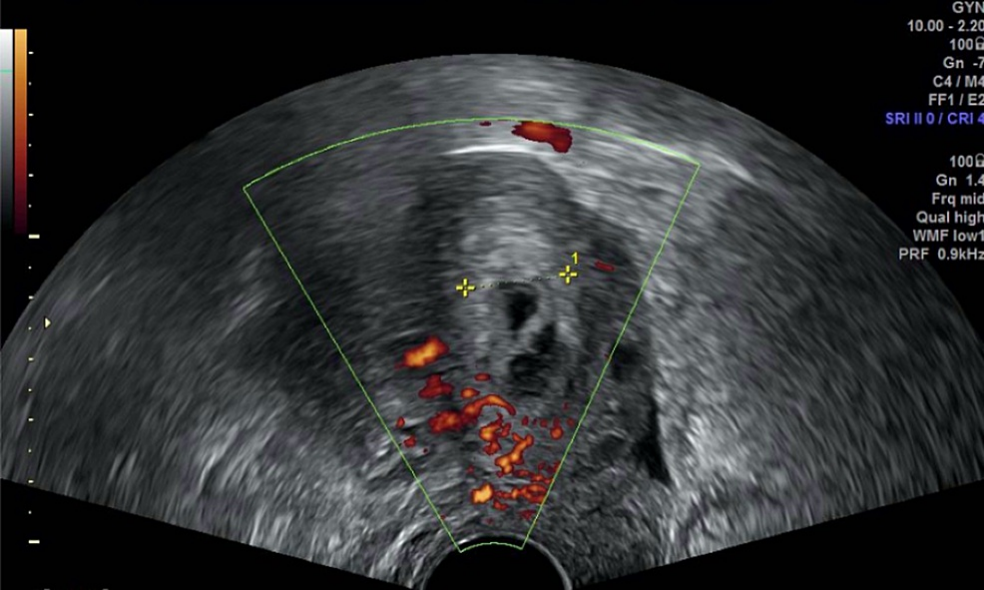
Transvaginal ultrasound (axial plane) of the residual trophoblastic tissue in resorption – mild peripheral vascularization

On the second postoperative day, the patient was discharged from the hospital. The clinical evolution was favorable. Follow-up included a weekly control of hCG, which became negative on the fourth week after discharge (49th day after diagnosis). Transvaginal ultrasound done on the first and second week after discharge still showed at the cervical level a nodular and cavitated image measuring 18 x 13 mm (Figure 6), with mild peripheral vascularization (Figure 7), suggesting residual trophoblastic tissue in resorption.

On the third week, the ultrasound was completely normal. Currently, the patient is clinically well under oral contraception.

## Discussion

Cervical pregnancy occurs when an embryo undergo nidation in the endocervical canal [3]. Any compromise in the capacity of the uterine cavity that prevents implantation in the endometrium could be a contributing factor [1]. The major risk factors include in vitro fertilization, endometrial injury caused by pelvic inflammatory disease or postsurgical trauma such as cesarean section or uterine curettage, history of abortions, intrauterine device use, and structural uterine anomalies [1]. Although it is a rare condition, nowadays, the incidence seems to be rising not only due to assisted reproductive technologies but also thanks to better access and accuracy of transvaginal ultrasound scanning [4,5].

The most common presenting symptom of cervical pregnancy is painless vaginal bleeding [5]. Taking into account the clinical and ultrasound findings, the differential diagnosis of cervical pregnancy should include low location intrauterine pregnancy such as cesarean scar pregnancy and even heterotopic pregnancy, incomplete abortion, and large nabothian cysts [5]. The ultrasound criteria to establish the diagnosis consists of the 1) absence of an intrauterine pregnancy, 2) presence of a gestational sac below the level of the internal cervical os, 3) absence of a sliding sign, and 4) presence of flowing blood around the gestational sac detected by Doppler [6]. Other additional signs include closed internal cervical os, an "8" or hourglass uterine shape (because of the ballooned cervical canal), endometrial decidualization, and the presence of embryonic structures in the ectopic gestational sac [7]. Regarding ultrasound features, cervical pregnancy can easily be misdiagnosed as scar pregnancy. The difference is that cervical pregnancy is located below the internal os, while scar pregnancy is located above, at the level of the cesarean scar incision. Cervical pregnancy can also be widely misdiagnosed as an ongoing abortion. The best way to distinguish the two situations is by the sliding sign, which means that when gentle pressure is applied on the cervix with the probe, the implanted cervical pregnancy does not slide, unlike the gestational sac of the abortion [1].

As a consequence of the rarity of the condition, therapeutic approaches are still mainly based on case series studies [3]. Thus, different from tubal ectopic pregnancy, the therapeutic protocols for cervical ectopic pregnancy have not yet been standardized, although there are general recommendations [8]. The treatment method should be based on the patient’s desire to preserve fertility, hemodynamic state, and gestational age, taking into account initial serum hCG level, crown-rump length, and the presence or absence of fetal heartbeat [9].

In a first trimester cervical pregnancy with a hemodynamically stable patient without severe bleeding, the preferred option is medical treatment [1]. Methotrexate, mifepristone, and misoprostol have been used to medically terminate a cervical pregnancy, usually in combination with interventional measures [5]. The most effective medical treatment is methotrexate, either by systemic administration in an intramuscular single-dose or multidose regimen and/or by local administration in an intra-amniotic injection. Failure of systemic methotrexate treatment occurs often when the serum hCG level is >10.000 mIU/mL, the crown-rump length is >10 mm, or fetal heartbeat is present [5]. In these cases, combined therapy with intra-amniotic methotrexate may increase the effectiveness of the treatment. In the presence of a heartbeat, an intra-amniotic administration of potassium chloride for embryocide or feticide is also recommended [7]. Some authors describe beneficial effects in aspiration and injection of approximately equal amounts of volume into the cavity when performing the intra-amniotic injection, so the internal pressure does not vary. This procedure prevents excessive bleeding [5]. This technique was used in our case during the intra-amniotic injection of methotrexate, although the procedure was ineffective but after all uneventful.

If the previous medical treatment fails or is contraindicated, a fertility-sparing surgery method could be adopted with the excision of the trophoblast via curettage/aspiration or surgical hysteroscopy [1]. However, due to the lack of smooth muscle tissue in the cervical region, there is a predisposition to massive bleeding during conservative surgery, which often requires an emergent hysterectomy to control hemorrhage [5]. Therefore, adjuvant measures to reduce blood loss post- or pre-procedure are recommended and include tamponade with a Foley balloon catheter or reduction of blood supply by vasopressor/prostaglandin cervical injections, cervical cerclage, surgical ligation of cervical/uterine/internal iliac arteries, and arterial embolization [2,7,8]. According to updated references, the use of uterine artery embolization in obstetric and gynecological emergencies has high efficacy and few complications [3,10]. Despite this, it is still an underused method, probably due to limited accessibility to interventional radiology with experience in this technique. In the present case, uterine artery embolization preceding the invasive procedure was essential to prevent the occurrence of massive hemorrhage and allow the safe surgical removal of the trophoblast. The ideal time for surgery after embolization has not yet been defined, ranging from immediately to hours or days later according to the literature [9].

Regardless of the conservative treatment used, the literature is unanimous regarding the need for follow-up until complete resolution, defined by negative serum hCG values (<5 mIU/mL). Ultrasound findings alone are not reliable as the inert trophoblast image takes several weeks to disappear [1]. Finally, a trustworthy contraceptive method for at least three months should be prescribed after methotrexate therapy regarding the teratogenic effect of the drug, classified through the FDA as pregnancy category X [3]. For future research, it would be important to define a comprehensive protocol for the management of cervical pregnancy.

## Conclusions

Emergency obstetricians and gynecologists should be aware that cervical ectopic pregnancies are rare but life-threatening if there is a misdiagnosis or delay in treatment. The treatment for cervical ectopic pregnancy should be individualized, mostly aiming for the preservation of reproductive potential if the patient has this desire and is hemodynamically stable. Methotrexate (systemic and/or local) is the most effective medical treatment. If methotrexate fails or is contraindicated, conservative surgery to remove the trophoblast can be done by curettage or surgical hysteroscopy. There is always a high risk of severe hemorrhage that may require hysterectomy; therefore, a procedure to reduce the bleeding risk before or after conservative surgery may be crucial. Uterine artery embolization can decrease morbidity, mortality, and the need for an emergent hysterectomy during an invasive procedure in patients with high risk of bleeding.
